# Oral Health Status and Cardinal Symptoms of Depression in the Old Order Amish: a cross-sectional study

**DOI:** 10.21203/rs.3.rs-10470389/v1

**Published:** 2026-09-30

**Authors:** Teodor T. Postolache, Abhishek Wadhawan, Kathy A. Ryan, Melanie L. Daue, Stefanie Malan-Müller, Aline Dagdag, Hina Makkar, Janna Volkov, Julie Kreyenbuhl, Andrew J. Hoisington, Lisa A. Brenner, Robert K. Ernst, Braxton D. Mitchell, Mark A. Reynolds

**Affiliations:** Mental Illness Research, Education and Clinical Centers; Saint Elizabeths Hospital; University of Maryland School of Medicine; University of Maryland School of Medicine; Hospital 12 de Octubre Research Institute (Imas12); University of Maryland School of Medicine; Northern Virginia Mental Health Institute, Department of Psychiatry, Falls Church, VA 22042, USA; Saint Elizabeths Hospital; Mental Illness Research, Education and Clinical Centers; Consortium for Research and Education (MVM-CoRE), Rocky Mountain Regional Medical Center; Departments of Psychiatry & Neurology, University of Colorado Anschutz Medical Campus, Aurora, Colorado, USA; University of Maryland School of Dentistry; Division of Endocrinology, Diabetes and Nutrition, Department of Medicine, University of Maryland School of Medicine, Baltimore, MD, USA; Department of Advanced Oral Sciences & Therapeutics, University of Maryland School of Dentistry, Baltimore, MD, USA

**Keywords:** Denture, dysphoria, hopelessness, anhedonia, depression, Old Order Amish

## Abstract

**Background:**

Although results have been mixed, possibly owing to high confounding heterogeneity within and across studies, multiple efforts suggest that tooth loss and periodontitis are associated with depression.

**Methods:**

We analyzed questionnaires on dental health and depression obtained as a component of a Wellness study in 4,288 Old Order Amish (mean age = 43.5 ± 16.8 years; 57% women), a population with relatively high genetic and lifestyle homogeneity. Specifically, we obtained questionnaires reporting the use of removable partial/complete dentures (false teeth) and cardinal symptoms of depression (dysphoria/hopelessness and anhedonia), and we analyzed their associations using multivariable logistic regression, with adjustment for age, sex, smoking status, and occupation (farmer vs. non-farmer).

**Results:**

We report statistically significant associations between dentures and current (but not “ever”) depression symptom ratings (p < 0.05). The most robust associations were between current anhedonia AND dysphoria/hopelessness and the presence of any dentures (versus no dentures) (OR 1.760 [1.131, 2.737], p = 0.012), and the presence of complete dentures (versus partial or no dentures) (OR 1.794 [1.008, 3.193], p = 0.047).

**Conclusions:**

In this relatively homogeneous population, denture use—particularly complete dentures—was associated with current anhedonia and dysphoria/hopelessness. A primary strength of the study is its relatively homogeneous population, with a low prevalence of smoking—an important confounding factor—and limited access to both prophylactic dental care and fluoridated water, which collectively enhance the reliability of the findings. Limitations include the cross-sectional design and reliance on self-reported data. These findings warrant replication in future longitudinal studies incorporating dental and clinical examinations, imaging, and laboratory biomarkers.

## 1. Introduction

Periodontal diseases are the most common dental plaque-induced inflammatory conditions localized to the gingiva or extending to the supporting structures of the teeth. Reversible inflammation of the gingiva without evidence of breakdown of the periodontium is characteristic of gingivitis [1–3] and, if untreated, can progress to periodontitis [4]. The hallmark of periodontitis is the irreversible breakdown of the connective tissue attachment to the root of the tooth and resorption of the alveolar bone, both primarily attributable to the immune and inflammatory response to bacterial pathogens [2]. Progressive periodontal destruction results in tooth mobility (loose teeth) and tooth loss [5–7]. The estimated prevalence of periodontitis was 42% of adults (≥ 30 years old) in the United States (US) based on data from the National Health and Nutrition Examination Survey [8]. Moreover, the prevalence of severe periodontitis was 7.8%, most prevalent among adults aged 65 years or older, smokers, Mexican American persons, and non-Hispanic Black individuals [8].

Infection, and in particular, periodontal disease is the most common cause of tooth loss [5, 6, 9]. Tooth loss is positively associated with both cardiovascular [10–12] and all-cause mortality [12]. Tooth mobility is strongly correlated with the degree of alveolar bone loss in periodontitis [13]. Tooth loss, and its prosthetic replacement by removable partial or complete dentures, thus represents a readily ascertainable marker of cumulative oral disease burden, and is the focus of the present study.

There is an elevated risk of periodontal disease in psychiatric patients, both inpatients [14–16] and outpatients [17]. Multiple studies have reported associations between tooth loss and depression [18–34]; however, other studies have not reached the same results [35–45]. Heterogeneous risk factors among individuals in these studies may have contributed to these discrepancies. These factors include varying associations between specific depressive symptoms and infections, co-existing substance use disorders, psychological trauma, pregnancy, medical conditions, medications, genetic susceptibility to depression linked with particular pathogen serotypes, and lifestyle differences.

For instance, alcohol use and smoking have been reported to be risk factors for both depression [46–48] and periodontitis [49–54]. In the present study, we decreased the number of confounding factors by examining a relatively homogeneous population of the Old Order Amish (OOA), who have a low prevalence of alcohol use and smoking [55, 56] and relatively similar socioeconomic status and lifestyles [57], and, likely, poor access to and use of prophylactic dental care across the community. Prosthetic tooth replacement (e.g., removable partial or complete dentures/false teeth) serves as a surrogate variable for tooth loss. We hypothesized that using a removable dental appliance would be positively associated with cardinal symptoms of depression, specifically anhedonia and/or dysphoria/hopelessness. We focused on these two symptoms because, according to the DSM-5, the presence of at least one of them—depressed mood (dysphoria/hopelessness) or markedly diminished interest or pleasure (anhedonia)—is required for the diagnosis of a major depressive episode, unlike the remaining symptoms [58]; moreover, anhedonia [59] and hopelessness [60] are each, independently, recognized risk factors for suicidal behavior.

## 2. Methods

### 2.1 Recruitment

The Amish Wellness Study is a community-based program of cardio-metabolic health carried out in the Lancaster, Pennsylvania Amish community that included a broad panel of lifestyle factors collected from family and medical history questionnaires and sleep and mood questionnaires. Our recruitment and study procedures in the Amish Wellness Study and the use of a modified Patient Health Questionnaire-2 (PHQ-2) have been previously described [61]. The PHQ-2 provides ratings of current and ever dysphoria/hopelessness and anhedonia and has been reported to have high sensitivity (83%–89.3%) and specificity (75.9%–92%) [62–64]. Self-reported measures of dental health were also obtained. The questions included on the modified PHQ-2 were: “during the last month have you often been bothered by feeling down, depressed, or hopeless?”, which represented “current” dysphoria/hopelessness, and “during the last month have you had little feelings of interest or pleasure?”, which represented “current” anhedonia. Participants were categorized as currently experiencing dysphoria/hopelessness AND anhedonia if they answered “yes” to both questions, or dysphoria/hopelessness OR anhedonia if they responded yes to either of the two symptoms on the PHQ-2. The questionnaires also asked whether the participant had experienced either of these symptoms in the past. If participants experienced any of these symptoms currently or in the past, they were categorized as “ever.” If participants answered “no” to both items on the PHQ-2, they were classified as having “no depressive symptoms.”

The question related to dental health that we analyzed for this study was: “Do you have false teeth?” (answers were “yes-full”, “yes-partial”, “no”, and “don’t know”). Information about smoking and occupation was obtained from the Amish Wellness Study Medical and Social History Inventory. For smoking adjustment, we used “ever” (yes, in the past OR yes, current use) versus “never” use, and included the following modalities of tobacco use: cigarettes, cigars, pipe, snuff, and chewing tobacco. For occupation, we transformed the multiple occupational categories on the original response categories of the Amish Wellness Study Medical and Social History Inventory into binary categories, “farmer versus non-farmer.” These original categories included: (a) housekeeping, housewife (both denote home-based domestic work and fall under the non-farmer category); (b) farming (dairy, plant, tobacco), horse keeping, field worker, farmer’s wife; (c) shopkeeping, marketing, gardening, waitress, cook, cleaning, livestock auction, caring, cashier; (d) clerk work, needlework, quilting, teaching, order buyer at hale sales, craft making, municipal waste management, shoe repair; (e) carpenter, smith, woodwork, mason, mechanic, welder, painting, sawmill, fencing, house framer; (f) construction, contractor, mobile home builder; (g) retired; and (h) other. The questionnaires were vetted during several feedback sessions by representatives of the Amish community (Amish liaisons with the Amish Clinic of the University of Maryland staff) and received their unanimous approval. The University of Maryland Baltimore Institutional Review Board approved the study protocol. To participate, members of the Amish community had to provide written consent after a full explanation of the study and having sufficient time to have all their questions answered. The research was conducted in accordance with the Declaration of Helsinki [Clinical Trial Number: not applicable; this is an observational study].

There were 6,230 Old Order Amish individuals living in Lancaster County, Pennsylvania, aged ≥ 18 years (57% women), who were recruited into the “parent” Amish Wellness Study between 2010 and 2019. The last participants in this set had their Wellness visit completed on 08/26/2019. Among the 6,230 participants enrolled in the Wellness study at the time of analysis, 1,709 (27.4%) had neither dental nor PHQ-2 questionnaires, 167 (2.7%) had dental only, and 66 (1.1%) had PHQ-2 only. Participants responding “don’t know” were excluded from analysis. The analyzed sample included 4,288 (68.8%) participants with both dental and PHQ-2 questionnaires. Figure 1 shows the Wellness study, the completion percentage for the dental and depression questionnaires, and the analyzed sample.

### 2.2 Statistical analyses

Analyses were performed using logistic regression models with dental variables (partial denture, complete dentures, or any denture) included as independent variables and symptoms of depression as dependent variables. Age, sex, smoking status (ever smoker versus never smoker), and occupational status (farmer versus non-farmer) were included as covariates in these analyses. For the comparison of partial denture versus no denture, edentulous participants (i.e., those with complete dentures) were excluded. Results are presented as odds ratios with 95% confidence intervals, and statistical significance was assessed using two-tailed p-values with α = 0.05. Statistical analyses were completed using the SAS 14.2 software package (Cary, NC, USA).

## 3. Results

The frequency of participants with depression symptoms according to the use of partial or complete dentures is provided in Table 1, and the associations between these variables, adjusted for age, sex, smoking status, and occupation, are presented in Table 2. A summary of these results is as follows:

### 3.1 Partial denture vs. no partial denture (excluding participants with complete dentures)

In these analyses, we excluded edentulous participants (those with complete dentures), and adjustment was done for the covariates age, sex, smoking, and occupational status. Individuals with a partial denture versus individuals with no dentures displayed a statistical trend toward higher odds of current anhedonia (OR 1.521, 95% CI [0.980, 2.361], p = 0.062), as well as ever anhedonia OR dysphoria/hopelessness (OR 1.262, 95% CI [0.964, 1.653], p = 0.091). There were no significant associations of partial denture with current dysphoria/hopelessness, current anhedonia AND dysphoria/hopelessness, or current anhedonia OR dysphoria/hopelessness (see Table 2).

### 3.2 Complete dentures vs. partial or no denture

In these analyses, covariates included age, sex, smoking, and occupational status. Individuals with complete dentures versus individuals with a partial or no denture had statistically significant higher odds of having current anhedonia AND dysphoria/hopelessness (OR 1.794, 95% CI [1.008, 3.193], p = 0.047). However, there were no significant associations with other depression-related variables (see Table 2).

### 3.3 Any denture (including both complete and partial dentures) vs. no denture

In these analyses, covariates included age, sex, smoking, and occupational status. In the entire sample, individuals with any denture versus individuals with no dentures had statistically significant higher odds of reporting current anhedonia (OR 1.560, 95% CI [1.062, 2.292], p = 0.024), as well as current anhedonia AND dysphoria/hopelessness (OR 1.760, 95% CI [1.131, 2.737], p = 0.012). Additionally, a statistical trend was identified for the association between any denture and ever anhedonia OR dysphoria/hopelessness (OR 1.238, 95% CI [0.982, 1.562], p = 0.071). There were no significant associations of any denture with other mood variables (see Table 2).

## 4. Discussion

### 4.1 Summary of key findings

As hypothesized, when compared with individuals without any dentures, participants with dentures were significantly more likely to report current anhedonia and combined current anhedonia AND dysphoria/hopelessness. When compared with participants without complete dentures (i.e., those with either no dentures or partial dentures), participants with complete dentures were significantly more likely to report current anhedonia AND dysphoria/hopelessness. A low-grade statistical trend was observed for individuals with partial dentures versus those with no dentures, and for those with any dentures versus those with no dentures, to endorse ever anhedonia OR dysphoria/hopelessness. Notably, the significant associations were confined to current, rather than “ever,” symptom ratings.

### 4.2 Strengths and limitations of the study

A primary strength of the study is its relatively homogeneous population. The OOA have a low prevalence of alcohol use and smoking [55, 56], relatively similar socioeconomic status and lifestyles [57], and limited access to prophylactic dental care and fluoridated water across the community, which collectively reduce confounding heterogeneity. In addition, the OOA are generally less exposed to the negative effects of intense evening light and low-wavelength light originating from televisions, computers, tablets, and mobile phone screens [65], and have a lower frequency of short sleepers [66]. However, in the Amish, current and lifetime stress levels do affect sleep [67], as do heritability (of wake-time [67], only after accounting for shared environment), chronotype [68], and seasonality [69]; these factors were not adjusted for in the present analyses.

Several limitations should be considered. The study is cross-sectional; therefore, we cannot ascertain a causal relationship between denture use and symptoms of depression. To measure symptoms of depression we did not use standardized structured diagnostic instruments, and for periodontal outcomes we did not corroborate responses with clinical notes, radiographs, or contemporaneous oral examinations. Data on sleep, diet, oral hygiene practices, and frequency of dental visits were not available. We also did not account for education and income; formal education is highly homogeneous in this population (schooling through the 8th grade for nearly all participants), and, while income may vary, lifestyle and healthcare access are relatively uniform owing to shared cultural norms. Importantly, we did not directly ask about tooth loss or its most common causes (periodontal disease, decay, and trauma), and dentures were used only as a surrogate for tooth loss. Consequently, “no denture” encompasses both individuals who have never lost teeth and those who lost teeth without replacement, which is a considerable shortcoming; this limitation is likely to blur differences in symptoms of depression between those with and without dentures, but is unlikely to create spuriously significant differences.

With respect to analysis and interpretation, the PHQ-2 is useful for screening and estimation but not for the clinical diagnosis of depression [63, 64]. Additionally, the expression of negative mood in the OOA is somewhat limited as a consequence of the OOA valuing gratitude and stoicism [57]. The OOA believe in accepting the hardships of daily life as God’s will (Gelassenheit), respond to adversity by yielding to divine providence, and often consider that divine intervention leads to the resolution of past symptoms [57]. Hence, the OOA are prone to underreport past symptoms once they are resolved, and a culturally based bias might have contributed to significant associations between dental status and current, but not past, symptoms of depression. Therefore, the generalizability of our findings to non-OOA populations may be limited. We expect that stronger effect sizes may emerge in less stoic populations, in particular for past symptoms; on the other hand, the higher heterogeneity of lifestyle could increase confounding and decrease statistical power.

### 4.3 Interpretation and implications in the context of the totality of evidence

Our results are consistent with the majority trend in previous studies [18, 20–27]. A meta-analysis of 14 studies by Liu et al. (2018) [18] confirmed a significant association between chronic periodontitis and “emotional disorder” (including depression and anxiety) (OR = 1.54, 95% CI 1.27–1.86). In a prospective study, one of the first of its kind, the “dental-to-mental” direction of causality was examined in outpatients with chronic and aggressive periodontitis to assess the impact of tooth loss on future depressive symptoms [19]. Tooth status of 310 patients was determined initially, and they were followed for 13 years for the subsequent development of depressive symptoms using the PHQ-9. Even after controlling for several covariates, including smoking status, age at baseline, and comorbid somatic illnesses, fewer teeth at the beginning of treatment were related to higher depressive symptom scores, establishing that tooth loss may be a risk factor for developing depression in individuals with periodontitis [19]. Similarly, a large US study indicated that adults with current depression, lifetime diagnosed depression, and lifetime diagnosed anxiety were significantly more likely to have had at least one tooth removed relative to depression- and anxiety-free individuals [28]. Compared with individuals with 0 teeth removed, the adjusted odds of being in the all-teeth-removed, 6–31-teeth-removed, or 1–5-teeth-removed categories were increased for individuals with current depression (adjusted odds ratio [AOR] = 1.44, 95% CI 1.11–1.86; AOR = 1.83, 95% CI 1.51–2.22; and AOR = 1.35, 95% CI 1.14–1.59, respectively) [28].

In contrast, no significant associations between depression and periodontal disease were reported in a meta-analysis [35], nor in cohort [36], case-control [37, 38], and cross-sectional studies [32, 39–42, 70]. It is possible that the heterogeneous distribution of certain confounding variables (substance use disorders, tobacco use, socioeconomic status, occupation, living environment and cultural practices, access to mental health and dental care services, and various amounts and modalities of fluoride intake) across studies contributed to differences in outcome. By evaluating a relatively homogeneous population (i.e., the OOA), we have minimized these potential confounders.

What are the potential mechanisms underlying the reported associations? Although progressive periodontitis often accounts for the majority of cases, other pathological pathways could contribute to tooth loss. For instance, advanced dental caries (decay) can induce endodontic infection and pain, which may result in extraction of the offending teeth. When untreated, a late-stage endodontic infection leads to apical periodontitis, a chronic inflammatory state of the peri-radicular tissues. Apical periodontitis shares important similarities with the microbial spectrum and immune-inflammatory response of periodontitis [71]; for example, it is also associated with significant serum elevations in inflammatory mediators such as CRP, IL-1, and IL-6 [72], which have been identified as predictors of cardiovascular events in humans [73]. However, a recent study using NHANES data found that depression is associated with tooth loss and periodontitis, but not with caries [74].

As noted earlier, tooth mobility and tooth loss exhibit a strong association with the severity of periodontitis, likely as a consequence of it [5–7, 13]. Periodontitis is associated with elevations in markers of inflammation [75], such as Type 1 cytokines, C-reactive protein [76–80], IL-6 [81], and IL-18 [78]. Furthermore, type 1 cytokines [IL-1β, IL-6, and tumor necrosis factor-α (TNF)] can be induced by periodontal pathogens and their products [82, 83]. Increasing evidence also links inflammation to symptoms of depression [84–90], and multiple non-human animal studies point toward a causal relationship between inflammatory mediators and depressive-like symptoms [91–96]. Research further supports the potential role of inflammation as a key mediator of the periodontitis–depression link [97–99]. Alterations in the oral microbiome could also provide a link with depression [100–102], potentially via upregulation of inflammation and impairment of barrier function (intestinal and blood-brain barrier [BBB]).

Sleep disturbance is also associated with both depression and tooth loss. Specifically, tooth loss and periodontitis are negatively associated with sleep duration and positively associated with perceived sleep insufficiency [103], and more generally with multiple disturbances of sleep duration and quality (e.g., obstructive sleep apnea) [104]. A spectrum of sleep disturbances—including insomnia, obstructive sleep apnea, hypersomnia, restless legs syndrome, aberrant sleep duration (both short and long), and eveningness chronotype orientation—are also associated with depression, as demonstrated by a systematic review and meta-analysis of longitudinal studies [105].

Translocation of periodontal pathogens from the oral cavity to the systemic and cerebral circulation and the central nervous system is another pathway connecting periodontal disease and mental health. Periodontal bacteria and their products can gain access to the circulation [106, 107], resulting in systemic dissemination of both bacterial products and the bacteria themselves. Invasive periodontal treatment may induce a temporary increase in pro-thrombotic/inflammatory mediators [108] and endothelial dysfunction within 24 hours after treatment [109], likely mediated by transient bacteremia due to trauma during treatment [110]; these changes may last up to 4 weeks post-treatment [108], leading to an increased risk of cardiovascular events during this period [111]. Moreover, even minor trauma such as toothbrushing can precipitate bacteremia episodes in patients with periodontitis [106, 112, 113]. Consistently, animal models of oral infection with Porphyromonas gingivalis, a keystone periodontal pathogen with demonstrated capability to translocate, have shown that inflammatory reactions due to this infection may be promoted in atheromatous lesions distant from the oral cavity [114–116]. Furthermore, the BBB is broken down via abnormal activation of matrix metalloproteinases by systemic injection of LPS [117, 118], which may allow molecular inflammatory products and phagocytes to enter the brain directly, ultimately and potentially leading to depressive mood and behavioral changes [119].

Periodontal disease [120] may also affect the gut microbiota [121], which may further contribute to mental health symptoms [122] through the gut-brain connection, as evidenced by animal [123–129], clinical [130], and imaging studies [131].

Poor oral health, particularly associated with tooth loss, could also lead to difficulty with communication, affecting other psychological and social aspects of human life [132] and worsening quality of life through diminished self-esteem and social status, impaired body image, and loss of chewing functionality [133–135]. Ill-fitting and painful dentition or dentures can cause difficulties with eating and speaking, and psychogenic incompatibility of prosthodontic replacement or denture dissatisfaction could also contribute to depressive symptoms [136, 137]. It is possible that patients with periodontitis experience social stigma from the negative effects of tooth loss on facial contour and aesthetics, as well as halitosis [138], which could precipitate or perpetuate anhedonia and/or hopelessness/dysphoria, thereby contributing to syndromic depression [139].

It is important to acknowledge that we chose to examine only two symptoms of depression (anhedonia and dysphoria/hopelessness) mainly for feasibility reasons. The team even considered simplifying further by asking only a single question about ever dysphoria/hopelessness OR anhedonia, but prior data did not support this additional simplification: the sensitivity for estimating the presence of major depression using a solitary question related to dysphoria/hopelessness is approximately 85–90%, and this sensitivity increases to 95% when an additional question related to anhedonia is added [62]. Both anhedonia and dysphoria/hopelessness are cardinal symptoms of depression, with at least one required for a defined major depressive episode, and both are well-recognized individual risk factors for suicidal behavior [140], beyond the presence of depression itself. These symptoms also allow application of the Research Domain Criteria to depression and suicide research, as both anhedonia and hopelessness can be individually modeled in animals [141]. Furthermore, anhedonia carries an individual negative prognosis regarding psychological, social, and physical functioning [142] and is linked with a more refractory response to antidepressant treatment [143].

From a clinical standpoint, our results suggest that it will likely be beneficial to foster bidirectional dialogue and collaboration between dentists and mental health providers.

### 4.4 Controversies raised by this study

Several considerations temper a causal interpretation of our findings. Because the study is cross-sectional and denture use serves only as a surrogate for tooth loss, the direction of any causal relationship between oral health status and symptoms of depression cannot be established. Moreover, increased stress [97, 144–148] could precipitate, exacerbate, perpetuate, and reactivate both periodontitis and depression and, together with other risk factors, could represent hidden “upstream” variables that may create false promises of causal associations—specifically, the hope that therapeutically targeting one domain will improve the other. It therefore remains possible that denture use is a marker of cumulative biological and psychosocial burden rather than a contemporaneous cause of depressive symptoms.

### 4.5 Future research directions

These findings warrant replication in future longitudinal studies incorporating dental and clinical examinations, imaging, and laboratory biomarkers. The apparent predominance of anhedonia over dysphoria/hopelessness as a contributor to our results requires further investigation; if the causal inferences underlying our associations were confirmed, periodontal prophylactic approaches could have the potential to prevent treatment refractoriness in depression among vulnerable individuals. More broadly, studies are needed to investigate in depth the reciprocal interactions between mental illness and periodontal disease, their treatments, and their overlapping immune, metabolic, and vascular associations.

## 5. Conclusions

In this relatively homogeneous population—characterized by a low prevalence of smoking and limited access to both prophylactic dental care and fluoridated water—denture use, and particularly the use of complete dentures, was associated with current anhedonia and dysphoria/hopelessness. These findings support a clinically meaningful link between oral health status and cardinal symptoms of depression and underscore the potential value of bidirectional collaboration between dental and mental health care. Given the cross-sectional design and the reliance on self-reported measures, the results warrant replication in longitudinal studies incorporating clinical dental and psychiatric examinations, imaging, and laboratory biomarkers.

## Supplementary Material

Supplementary Files

This is a list of supplementary files associated with this preprint. Click to download.

- SuprTable1b.docx
- SuprTable2b.docx

## Figures and Tables

**Figure 1:**
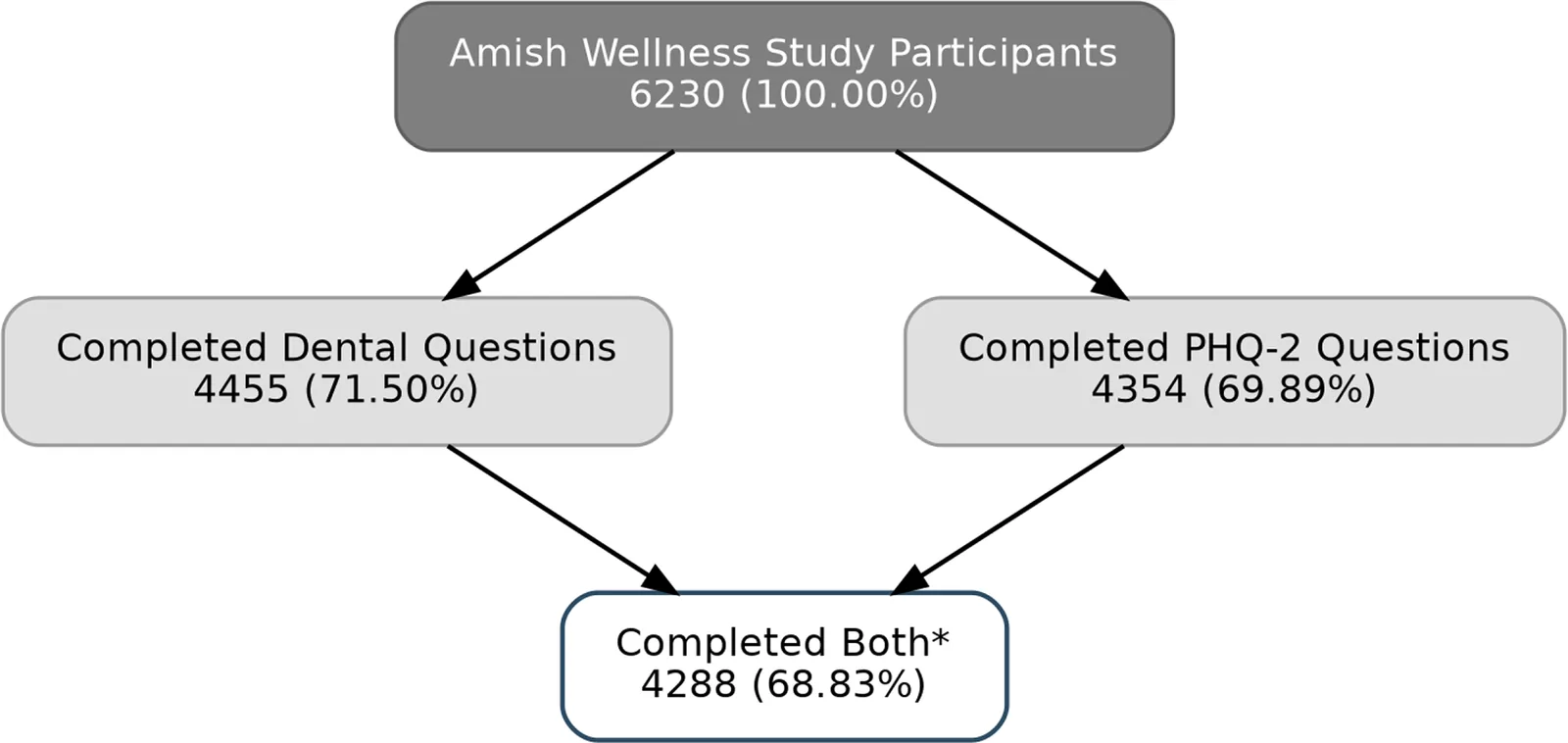
Wellness Study: completion percentage for the dental and depression questionnaires and the analyzed sample of completers of both questionnaires. Of 6,230 participants enrolled in the “parent” Wellness study at the time of the data freeze, 167 (2.7%) had dental questionnaires only, 66 (1.1%) had PHQ-2 questionnaires only, and 1,709 (27.4%) had neither dental nor PHQ-2 questionnaires. Our study included the 4,288 participants who completed both the PHQ-2 and dental questionnaires. *Indicates the study/analysis sample.

**Table 1:** Frequency of participants with depression symptoms according to dental status (partial and complete dentures).

Variables	Final Sample (N = 4,288)	All teeth intact (n, % = 3,389, 76.1%)	Partial denture, present (n, % = 589, 14.8%)	Complete denture, present (n, % = 425, 9.9%)
Age, mean ± SD, years	43.5 ± 16.8	39.1 ±14.4	54.1 ±15.6	62.4 ± 14.8
Current dysphoria/hopelessness, present, n (%)	381 (9.0%)	281 (6.7%)	51 (9.3%)	37 (8.7%)
Current anhedonia, present, n (%)	219 (5.1%)	159 (3.8%)	33 (5.9%)	20 (4.6%)
Current anhedonia AND dysphoria/hopelessness, present, n (%)	158 (3.7%)	115 (2.7%)	22 (4.0%)	18 (4.1%)
Current anhedonia OR dysphoria/hopelessness, present, n (%)	442 (10.3%)	325 (7.7%)	62 (11.1%)	39 (8.9%)
Ever anhedonia OR dysphoria/hopelessness, present, n (%)	727 (17.0%)	536 (12.9%)	99 (18.2%)	71 (16.7%)

Percentages for the “All teeth intact,” “Partial denture,” and “Complete denture” columns are computed relative to the final sample (N = 4,288); symptom percentages within each column are computed relative to that column’s denominator.

**Table 2:** Associations between dentures (independent variables) and cardinal symptoms of depression (dependent variables), adjusted for age, sex, smoking status, and occupation (farmer versus non-farmer).

Variables	Partial denture vs. no denture — OR [95% CI]	Complete dentures vs. partial or no denture — OR [95% CI]	Any denture (complete and partial) vs. no denture — OR [95% CI]
Current dysphoria/hopelessness	1.259 [0.880, 1.801], p = 0.208	1.148 [0.754, 1.748], p = 0.521	1.281 [0.942, 1.742], p = 0.115
Current anhedonia	1.521 [0.980, 2.361], p = 0.062^	1.269 [0.747, 2.157], p = 0.379	1.560 [1.062, 2.292], p = 0.024 *
Current anhedonia AND dysphoria/hopelessness	1.519 [0.896, 2.573], p = 0.121	1.794 [1.008, 3.193], p = 0.047 *	1.760 [1.131, 2.737], p = 0.012 *
Current anhedonia OR dysphoria/hopelessness	1.298 [0.934, 1.804], p = 0.120	1.001 [0.668, 1.499], p = 0.997	1.253 [0.940, 1.670], p = 0.125
Ever anhedonia OR dysphoria/hopelessness	1.262 [0.964, 1.653], p = 0.091^	1.065 [0.777, 1.461], p = 0.695	1.238 [0.982, 1.562], p = 0.071 ^

Partial denture vs. no denture comparison excludes subjects with complete dentures.

* Statistical significance (p < 0.05).

^ Statistical trend (p < 0.10).

## Data Availability

The datasets generated and/or analyzed during the current study are not publicly available due to the absence of Amish participant consent for data sharing. Summary and aggregate data rather than individual data could be made available upon reasonable request emailed to the corresponding author.

## Abbreviations

CI: confidence interval
DSM-5: Diagnostic and Statistical Manual of Mental Disorders, Fifth Edition
IRB: Institutional Review Board
MIRECC: Mental Illness Research, Education and Clinical Center
MVM-CoRE: Military and Veteran Microbiome: Consortium for Research and Education
NORC: Nutrition Obesity Research Center
OOA: Old Order Amish
OR: odds ratio
PHQ-2: Patient Health Questionnaire-2
SD: standard deviation
VISN: Veterans Integrated Service Network
